# Drug rash with eosinophilia and systemic symptoms syndrome masquerading as a lymphoproliferative disorder in a young adult on immunosuppressive therapy for rheumatoid arthritis: a case report

**DOI:** 10.1186/s13256-022-03526-0

**Published:** 2022-09-05

**Authors:** Elise Hyser

**Affiliations:** Department of Internal Medicine, AMITA Health St. Francis Hospital, 355 Ridge Avenue, Evanston, IL 60202 USA

**Keywords:** DRESS syndrome, Immunogenetics, Lymphoproliferative disorder, Steroids

## Abstract

**Background:**

This case reveals a novel presentation of drug rash with eosinophilia and systemic symptoms syndrome that mimics a lymphoproliferative disorder. The heterogeneous clinical presentation of drug rash with eosinophilia and systemic symptoms syndrome gives rise to a broad differential diagnosis that includes a multitude of infectious, inflammatory, and autoimmune conditions. This patient was diagnosed with drug rash with eosinophilia and systemic symptoms syndrome 4 weeks after starting sulfasalazine and 5 weeks after starting hydroxychloroquine for rheumatoid arthritis. Both of these medications have been shown to cause drug rash with eosinophilia and systemic symptoms syndrome, albeit more rarely in the context of hydroxychloroquine. This patient’s history, physical examination, and workup illuminate a case of drug rash with eosinophilia and systemic symptoms syndrome that masquerades as a lymphoproliferative disorder despite its adherence to the RegiSCAR criteria.

**Case presentation:**

A 22-year-old African-American female with an atopic history and rheumatoid arthritis presented for evaluation of a rash, unremitting fevers, and syncope. She was found to have drug rash with eosinophilia and systemic symptoms syndrome. A syncope workup was unremarkable. Computed tomography of the chest/abdomen/pelvis confirmed extensive lymphadenopathy and revealed a small right pleural effusion (Fig. 5). These imaging findings accompanied by fevers and a rash in the setting of eosinophilia, leukocytosis, and transaminitis led to the clinical suspicion for drug rash with eosinophilia and systemic symptoms syndrome. Steroids were subsequently initiated. Broad-spectrum antibiotic therapy was implemented to cover for possible skin/soft tissue infection due to initial paradoxical worsening after discontinuation of the culprit drugs. Lymph node biopsy ruled out a lymphoproliferative disorder and instead demonstrated necrotizing lymphadenitis. An extensive infectious and autoimmune workup was noncontributory. Clinical improvement was visualized, antibiotics were discontinued, and she was discharged on a steroid taper.

**Conclusion:**

This case reflects how drug rash with eosinophilia and systemic symptoms syndrome can masquerade as a lymphoproliferative disorder. Additionally, it highlights the extent to which rapid identification and treatment optimized the patient’s outcome. It calls into question how immunogenetics may factor into a patient’s susceptibility to acquire drug rash with eosinophilia and systemic symptoms syndrome. This case is unique because of the early onset of visceral organ involvement, the type of internal organ involvement, the hematopoietic features, and the lymphadenopathy associated with a disease-modifying antirheumatic drug.

## Introduction

Drug rash with eosinophilia and systemic symptoms (DRESS) syndrome constitutes a severe hypersensitivity reaction to a drug that clinically manifests with skin eruption, fever, hematological abnormalities, and internal organ involvement that can progress to multiorgan failure [1, 2]. Outcomes can be grim in the absence of timely recognition and treatment, and the condition carries a mortality rate of up to 10% [1, 2]. Factors that contribute to delayed diagnosis and treatment include the heterogeneous presentation, the prolonged latency period, and the broad differential [1]. While a multitude of medications can represent the inciting cause, common culprits include antibiotics, allopurinol, and antiepileptic agents [3]. DRESS syndrome is a clinical diagnosis. The RegiSCAR group, a multinational registry of severe cutaneous adverse reactions, formulated a set of clinical criteria that are widely accepted by the medical community ([1, 4], Table 1).

**Table 1:**
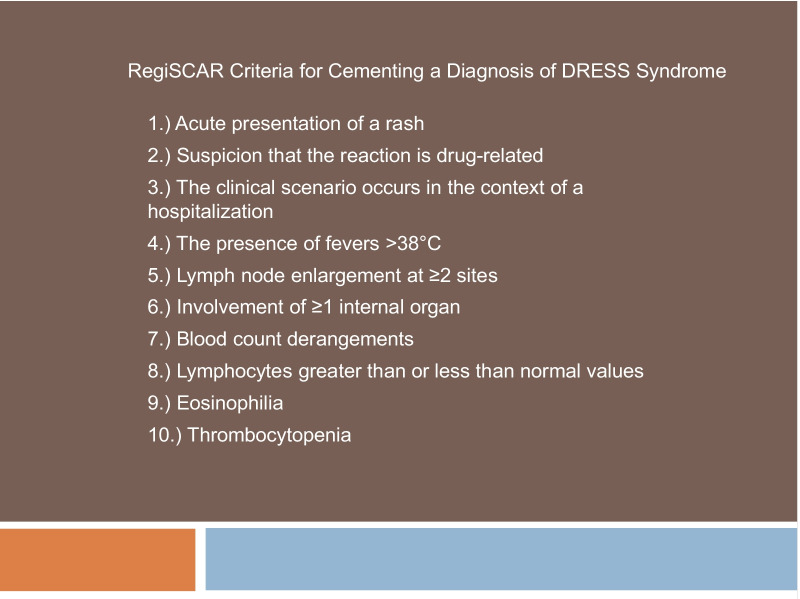
A set of clinical criteria utilized to diagnose DRESS syndrome

At least three of the seven features must be present, and a score above 5 solidifies the diagnosis.

Differentials to consider alongside this condition include Stevens–Johnson syndrome (SJS), toxic epidermal necrolysis (TEN), acute generalized exanthematous pustulosis (AGEP), systemic lupus erythematosus, lymphomas, hypereosinophilic syndromes, and Kikuchi disease [5–9]. In this case, DRESS syndrome masqueraded as a lymphoproliferative disorder. Lymph node biopsy ruled out an oncological process. Hydroxychloroquine or sulfasalazine are potential etiologies of DRESS syndrome. Although the clinical features of DRESS syndrome often evolve sequentially and mimic other diseases with the heterogeneous presentation, this patient fit the criteria for diagnosis based on the RegiSCAR criteria from initial presentation ([3], Table 1). Early clinical suspicion, discontinuation of the presumed culprit medication, and prompt delivery of steroids yielded a positive outcome [1, 2]. Cyclosporine can serve as a second-line therapy in steroid-refractory cases [2]. Defining the role of immunogenetics in expanding our understanding of the acquisition of DRESS syndrome is an important task.

## Case report

A 22-year-old African-American female with environmental allergies and rheumatoid arthritis presented with a rash, fevers to *T*_max_ 39.4 °C, and a syncopal episode. The syncopal event occurred the night before admission when she arose from bed to obtain water after feeling dehydrated. She denied prodromal chest pain, palpitations, dyspnea, nausea, or dizziness.

The day prior to admission, she developed facial swelling and a rash 1 hour after taking hydroxychloroquine. She denied associated dyspnea, wheezing, lip or tongue swelling, throat tightness, or vomiting. She applied triamcinolone ointment with no relief. Of note, she began hydroxychloroquine 5 weeks prior for rheumatoid arthritis. She started sulfasalazine 4 weeks prior, yet her rheumatologist discontinued it 6 days before admission following a pruritic lower-extremity rash that erupted within 3 hours of administration. She was on methotrexate 4 months prior, which she stopped because of nausea. She previously had poorly controlled rheumatoid arthritis that led her rheumatologist to prescribe these disease-modifying antirheumatic drugs. She did not tolerate the medications as evidenced by her reactions, although they relieved her joint pain from rheumatoid arthritis.

The patient reported generalized weakness coupled with night sweats for the past 2 weeks, and spiked fevers up to 39.4 °C the week preceding admission. She noticed a dry cough and a tender lymph node in the back of her neck a few days earlier. During the past 6 months, she had an unintentional 15-pound weight loss. She had two episodes of small-volume hematuria a few weeks ago. Family history is significant for rheumatoid arthritis in her mother and ovarian cancer in her great aunt. She denied recreational drug use. She reported eczema and sensitivity to multiple hair/skin products.

**Fig. 1:**
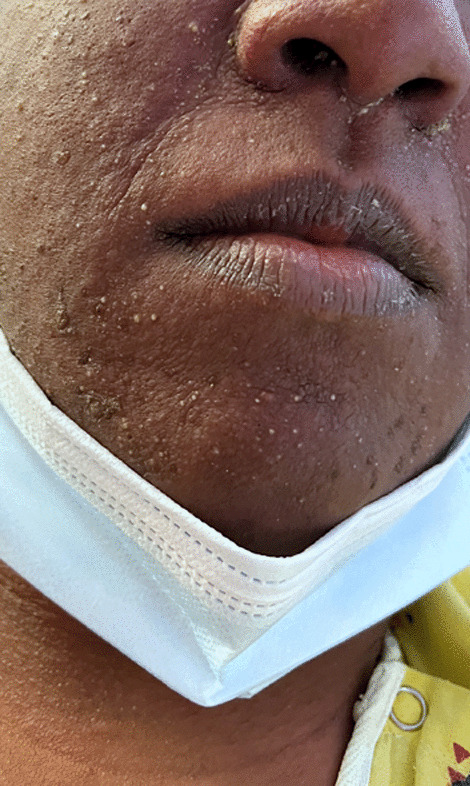
The appearance of the morbilliform rash involving the face on day 1 of the hospitalization

**Fig. 2:**
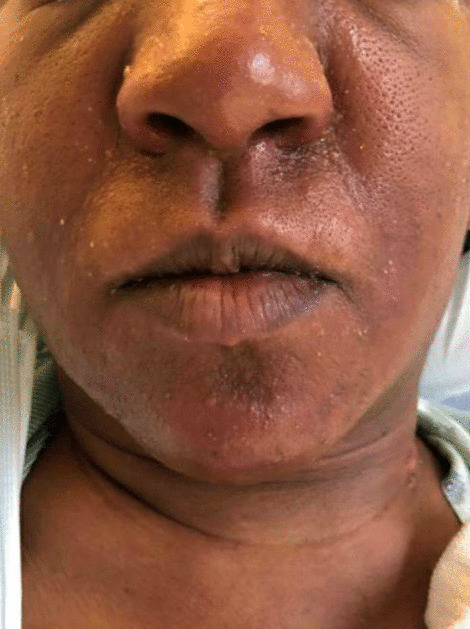
The appearance of the morbilliform rash involving the face on day 2 of the hospitalization

**Fig. 3:**
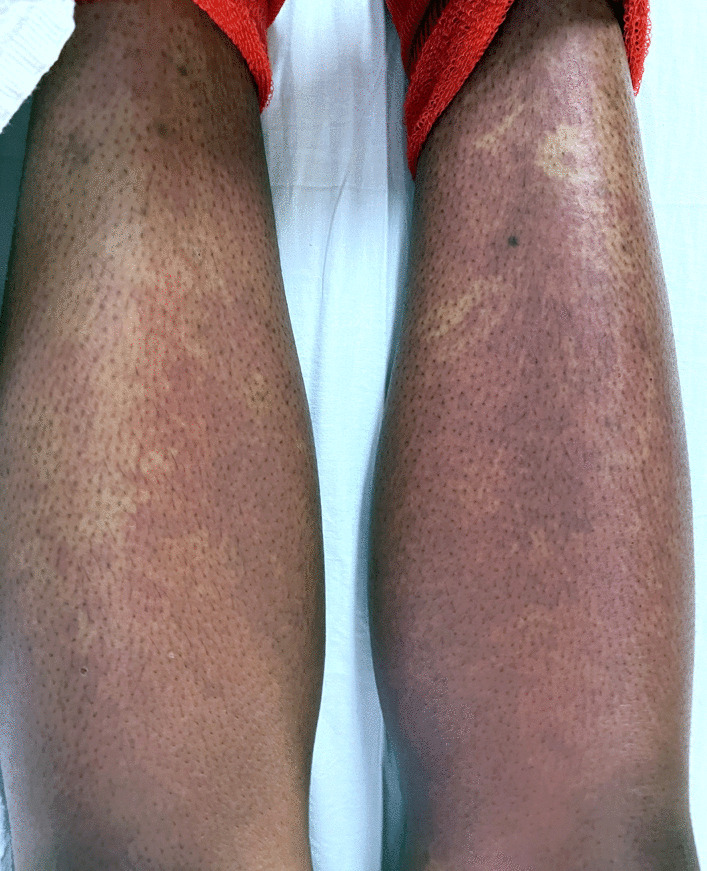
The appearance of the morbilliform rash involving the bilateral legs on day 2 of the hospitalization

**Fig. 4:**
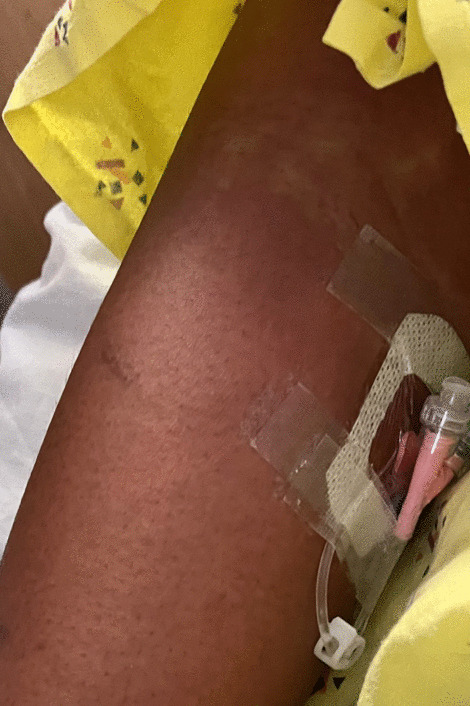
The appearance of the morbilliform rash involving the upper extremity on day 2 of the hospitalization

Initial vitals gathered in the emergency department were as follows: blood pressure 103/62 mmHg, pulse 119 beats per minute, temperature 38 °C, respiration rate 32 breaths per minute, and oxygen saturation 100% on room air. Physical examination showed an ill-appearing tachycardic female with a diffuse morbilliform erythematous rash extending from the face down to the lower extremities (Figs. 1, 2, 3 and 4). Mucous membranes were clear. Facial swelling was prominent in the bilateral cheeks and nose, sparing the lips and tongue. Scattered nontender mobile lymph nodes were palpated in the subsegmental, cervical, supraclavicular, bilateral axillary, and bilateral inguinal regions, with the largest lymph node measuring 1.5 × 1.5 × 1.5 cm in the posterior cervical region.

Labs revealed a transaminitis [aspartate transaminase (AST) 99 IU/L, alanine transaminase (ALT) 102 IU/L, alkaline phosphatase 205 IU/L], leukocytosis (13,100 cells/μL on admission, 36,600 cells/μL at its peak), and eosinophilia (1500 cells/μL). These results, coupled with administration of sulfasalazine and hydrochloroquine in the past 8 weeks, a morbilliform rash, fevers > 38 °C, diffuse lymphadenopathy, and internal organ involvement (pleural effusion), led to the diagnosis. While the transaminitis, hematopoietic abnormalities, and lymphadenopathy were detected on admission, the fevers began to spike and the morbilliform rash became more apparent on hospital day 2. The sulfasalazine and hydroxychloroquine were held from the moment she was admitted. A paradoxical worsening of symptoms was noted after the culprit medications were discontinued. Steroids were started from hospital day 1.

The fevers to *T*_max_ 39.4 °C in the setting of worsening facial swelling, skin erythema, leukocytosis, and procalcitonin of 4.45ng/mL on hospital day 2 prompted an infectious workup. Vancomycin and piperacillin/tazobactam were started to cover for possible skin/soft tissue or line infection.

An extensive infectious workup was sent, which was negative with the exception of an incidentally elevated mycoplasma IgM (2.21g/L). Other components of the infectious workup that were sent included human immunodeficiency virus (HIV) antigen/antibody, hepatitis panel, Epstein–Barr virus (EBV) and cytomegalovirus (CMV) polymerase chain reaction (PCR), urinalysis, blood culture, streptococcal and* Legionella* urine antigens, serum cryptococcal antigen, urine histoplasma/urine blastomyces antigens, *Coccidioides immitis* antibody, Fungitell 1,3-β-d-glucan, acid-fast bacilli (AFB) smear, ultrasound abdomen, and ultrasound head/neck.

**Fig. 5:**
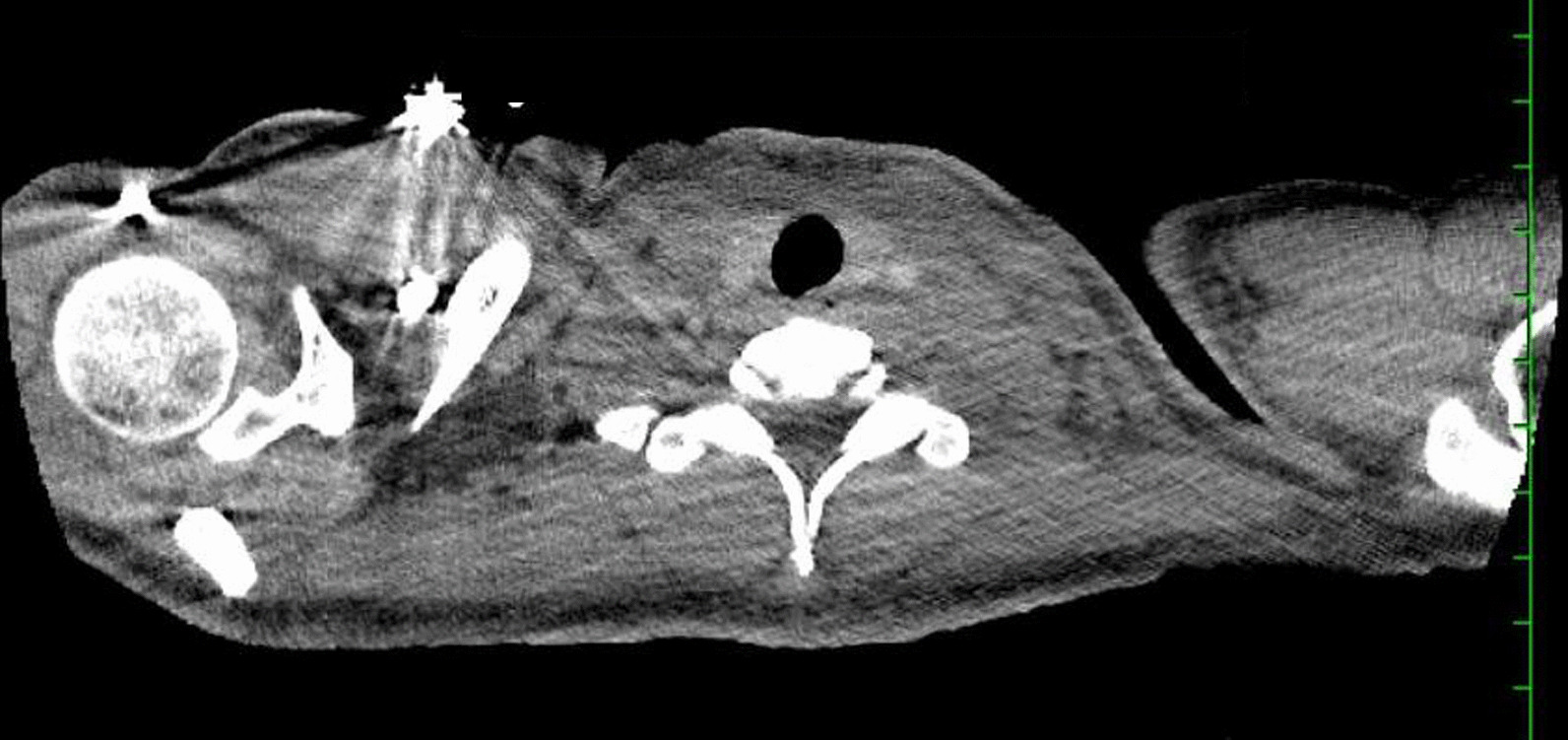
The diffuse lymphadenopathy noted on CT abdomen/pelvis from admission

Results of an autoimmune workup, most of which were obtained on hospital day 3, were noncontributory: C-reactive protein 5.8mg/L, erythrocyte sedimentation rate 31mm/hr, cyclic citrullinated peptide >250IU/mL, rheumatoid factor 15.5IU/mL, C3 level 101g/L, and C4 level 17g/L. Serum protein electrophoresis, antinuclear antibody, and antineutrophilic cytoplasmic antibody were negative. A syncope workup was unremarkable, which included orthostatic vitals, cardiac enzymes, electrocardiogram (EKG), and echocardiogram. Computed tomography (CT) of chest/abdomen/pelvis confirmed extensive lymphadenopathy and illuminated a small right pleural effusion (Fig. 5). Lymph node biopsy performed on hospital day 5 ruled out a lymphoproliferative disorder and exposed necrotizing lymphadenitis. The rash and fever curve improved with steroids, and the patient was discharged on a 7-week steroid taper. Antibiotics were stopped prior to discharge.

## Discussion

DRESS syndrome is a rare yet serious condition that can manifest in both children and adults as a morbilliform cutaneous rash accompanied by fevers, lymphadenopathy, hematological abnormalities, and visceral organ involvement [10]. It was historically referred to as phenytoin hypersensitivity syndrome, yet the name was changed when a multitude of additional culprit medications were elucidated [10]. While research seeks to further explain the pathogenesis, DRESS syndrome appears to be attributed to a delayed immunological reaction to the causative agent, a transient state of immunosuppression, and potential reactivation of latent herpes virus infections [10]. Visceral organ failure is often the cause of death in patients who succumb to the condition, and fulminant hepatitis with concomitant hepatic necrosis is commonly observed in these cases [3, 11].

Immunogenetic factors can raise a patient’s risk of developing DRESS syndrome [12]. Certain human leukocyte antigen (HLA) haplotypes have been linked to it [12]. For instance, there is a heightened susceptibility to reactions to carbamazepine in patients with the HLA-B*1502 variant [12]. Additionally, allopurinol can induce reactions among patients harboring the HLA-B*5801 allele [12]. Interleukin (IL)-17 is overexpressed in DRESS syndrome, including IL-17E, which can further raise circulating eosinophils, eotaxin, IL-4 level, IL-5 level, and IgE, thereby enhancing the eosinophilic immune response [12]. Genetic variants of IL-17 pathways can influence the development of atopy [13]. This patient had environmental allergies, eczema, and sensitivity to multiple hair/skin products. It would not be surprising if dysregulation of IL-17 rendered her susceptible to developing DRESS syndrome.

DRESS syndrome is recognized as a clinical diagnosis, which can be facilitated by the RegiSCAR criteria ([1], Table 1). A score above 5 solidifies the diagnosis [1]. This patient had a score of 7 for hospitalization, reaction suspected to be drug-related, acute rash, fever > 38 °C, enlarged lymph nodes at multiple sites, involvement of at least one internal organ (lung in the form of a pleural effusion), and blood count abnormalities ([1], Table 1). The lymphocyte toxicity assay (LTA) is a new diagnostic modality [14]. In LTA, the patient’s lymphocytes are isolated from the peripheral blood sample and incubated with the suspected culprit drug in the presence of a source of cytochrome p450 monooxygenase activity. [14]. Enhanced cell death correlates with a patient’s risk of having a hypersensitivity reaction to the drug used in the test [14]. This case did not warrant use of the LTA. The RegiSCAR criteria cemented the diagnosis.

The differential for DRESS syndrome includes SJS, TEN, AGEP, lupus, lymphomas, hypereosinophilic syndromes, and Kikuchi disease [5–9]. DRESS syndrome, SJS, and TEN all represent forms of severe cutaneous drug-induced eruptions [12]. While DRESS syndrome is associated with a morbilliform rash, SJS and TEN are severe mucocutaneous eruptions marked by diffuse erythema, blistering, and desquamation of the skin along with two or more other mucosal surfaces [3]. SJS and TEN are treated by removing the inciting drug and implementing supportive care measures [3].

AGEP is distinguished from DRESS syndrome by its nonfollicular small pustules surrounded by edema and erythema [5, 15]. This, coupled with epidermal acanthosis, spongiosis, and microvesicle and pustule formation on biopsy, makes the diagnosis [5, 15]. Immunophenotypical features identify DRESS syndrome: cutaneous effector lymphocytes represent a large quantity of the polyclonal CD8^+^ granzyme B^+^ T lymphocytes [15, 16]. AGEP and DRESS syndrome are managed by withdrawal of the culprit drug and initiation of steroids [5, 15, 16]. Lupus’ heterogeneous presentation requires satisfaction of 4/11 American College of Rheumatology criteria [6]. Treatment modalities for lupus include nonsteroidal anti-inflammatory drugs (NSAIDs), antimalarials, steroids, and immunosuppressive agents [6].

Extensive lymphadenopathy made it necessary to rule out a lymphoproliferative disorder. Cutaneous T-cell lymphomas are the most common type of cutaneous lymphoma, including mycosis fungoides, Sézary syndrome, cutaneous CD30^+^ T-cell lymphoproliferative disorders, and primary cutaneous peripheral T-cell lymphoma [7]. Lymph node biopsy showed a reactive process rather than malignancy. Hypereosinophilic syndromes are defined by eosinophilia > 1.5 × 10^9^ cells/L for > 6 consecutive months, eosinophil-induced organ damage, and exclusion of allergic, parasitic, and malignant etiologies for hypereosinophilia [8]. Kikuchi disease, or histiocytic necrotizing lymphadenitis, is a rare etiology of lymphadenopathy [9]. It is a benign, self-limited disease presenting with fever, fatigue, and night sweats, sometimes accompanied by joint pain and a rash [9]. Diagnosis requires analysis of clinical and histopathologic characteristics, subdivided into three types: early proliferative, necrotizing, and xanthomatous [9].

This particular case of DRESS syndrome was novel owing to multiple aspects of the presentation and clinical course. It has been discovered that internal organ involvement frequently impacts the hematopoietic, hepatic, and renal systems [17]. While the patient displayed evidence of hematopoietic involvement via leukocytosis and eosinophilia, along with hepatic involvement given the transaminitis, she also showed signs of pulmonary involvement given the cough and dyspnea in the setting of a new pleural effusion. Visceral organ involvement is typically a delayed manifestation of DRESS syndrome that can occur weeks to months after the appearance of the skin rash [17]. In this patient, the leukocytosis, transaminitis, and pleural effusion were noted on day 1 of the hospital stay, and the eosinophilia became evident on hospital day 2. In fact, the early organ involvement associated with this case facilitated the rapidity of the diagnosis by helping to fulfill elements of the RegiSCAR criteria.

Some of the hematologic manifestations of this patient’s case highlight the novelty of the clinical presentation. Although cytopenias are infrequently reported in conjunction with DRESS syndrome, this patient’s hemoglobin (Hb) dropped to 8.7 g/dL during the hospital stay despite having a baseline Hb of 12.4 g/dL [17]. Of note, no evidence of bleeding was found. Another unique hematological feature in this case was the lymphadenopathy in the absence of an association with nonsteroidal anti-inflammatory medications (NSAIDs) [17]. While it is known that lymphadenopathy is observed in 54–71% of cases of DRESS syndrome, it is more commonly witnessed with NSAIDs [17]. In contrast, this presentation involved diffuse lymphadenopathy on presentation, despite the lack of association with NSAID use.

In up to 80% of patients with DRESS syndrome, a concomitant finding of human herpesvirus reactivation is noted [17]. A few of the commonly encountered human herpesviruses implicated in DRESS syndrome include human herpesvirus (HHV)-6, EBV, HHV-7, and CMV [17]. Although the infectious workup completed included EBV and CMV PCR, viral detection might not be present until 3–5 weeks following symptom onset [17]. Since the human herpesviruses are ubiquitous, there is a possibility that reactivation of one of them was involved in this case, despite the fact that this was not pinpointed in the workup.

One of the feared complications that needed to be investigated in this case was the possibility of cardiac involvement. One retrospective analysis detected a prevalence of cardiac involvement among 19.1% of patients diagnosed with DRESS syndrome [17]. Some of the most severe cases display evidence of hypersensitivity myocarditis, acute necrotizing eosinophilic myocarditis, and restrictive cardiomyopathy [17]. However, patients with cardiac involvement may experience symptoms that could be shared by other organ pathologies, such as shortness of breath, hypotension, chest pain, and tachycardia [17]. For this reason, it was critically important to rule out the presence of any structural heart disease in the syncope workup, which returned negative. In some instances, cardiac manifestations can be delayed, which necessitates close follow-up for the patient.

## Conclusion

Although DRESS syndrome represents a rare condition, it is an important diagnosis to make as delayed treatment compromises outcomes. Early removal of the culprit medication, either sulfasalazine or hydroxychloroquine here, coupled with early institution of steroids optimized the clinical course. The high RegiSCAR score from presentation cemented the diagnosis. Immunogenetic factors can predispose certain individuals to acquiring DRESS syndrome [12]. While immunogenetic profiling was not performed on this patient, it is possible that her atopic history rendered her susceptible to developing DRESS syndrome [12]. Genetic variants of IL-17 pathways can impact the presence of atopy, and dysregulation of IL-17 can predispose individuals to acquiring DRESS syndrome [13]. Further research is needed to elucidate the complexity of immunogenetics in influencing a patient’s risk of being diagnosed with DRESS syndrome.

Several differentials aside from infection were necessary to ponder here. A lymphoproliferative disorder was a key differential to exclude given the extensive lymphadenopathy on CT in the context of fevers, fatigue, night sweats, and unintentional weight loss. Lymph node biopsy was consistent with necrotizing lymphadenitis secondary to DRESS syndrome, rather than a malignant process. Even though steroids are the treatment of choice, cyclosporine can be delivered in steroid-refractory cases [2]. In fact, one retrospective case–control study performed at Massachusetts General Hospital found cyclosporine to decrease disease progression and improve clinical/laboratory markers [2]. While autoimmune sequelae such as diabetes and thyroiditis can follow cases of DRESS syndrome, this patient fortunately did not suffer any long-term complications [18]. Features of this case that rendered it unique include the early onset of internal organ involvement, the type of visceral organ involvement, the hematopoietic profile, and the lymphadenopathy associated with a disease-modifying antirheumatic drug.

## Data Availability

Not applicable.
